# Occurrence and Prevention of Delayed Autonomous Selfing in *Salvia umbratica* (Lamiaceae)

**DOI:** 10.3389/fpls.2021.635310

**Published:** 2021-07-26

**Authors:** Han-Wen Xiao, Yan-Bo Huang, Yu-Hang Chang, Yun Chen, Richard J. Abbott, Yu-Kun Wei, Yong-Peng Ma

**Affiliations:** ^1^Shanghai Chenshan Botanical Garden, Shanghai, China; ^2^Eastern China Conservation Center for Wild Endangered Plant Resources, Shanghai, China; ^3^Yunnan Key Laboratory for Integrative Conservation of Plant Species With Extremely Small Populations, Kunming Institute of Botany, Chinese Academy of Sciences, Kunming, China; ^4^School of Biology, University of St Andrews, St Andrews, United Kingdom

**Keywords:** delayed self-pollination, pollen-limitation, recurving styles, seed set, *Salvia*, Lamiaceae, floral longevity, resource use

## Abstract

Delayed autonomous selfing (DAS) provides reproductive assurance under conditions of pollinator and/or pollen-limitation. Few plant species have been investigated to determine if DAS is terminated when a flower is sufficiently pollinated by a pollen vector, thereby saving plant resources for other purposes. We examined this possibility in bumblebee-pollinated *Salvia umbratica*. We first showed that DAS resulting in high fruit set (100%) and seed set (>80%) per flower occurred in the absence of insect pollinators by means of style recurvature and was completed in 94% of flowers 72 h after they opened. In contrast, in flowers pollinated immediately after opening, DAS was prevented by corollas dropping away before styles recurve toward the upper thecae. We next showed that hand-pollination of flowers immediately after they opened resulted in high fruit set (100%) and seed set (>80%) when 5–10 pollen grains or more were deposited on their stigmas, whereas fruit set and seed set were reduced to 45.00 and 22.50%, respectively, when pollen loads were reduced to 1–3 pollen grains. Finally, we showed that on average single pollinator visits deposited 26 pollen grains on stigmas of flowers that had just opened, which is more than enough to ensure high fruit and seed set. Our results indicate that flower longevity is highly correlated with the pollinator environment and female fitness of *S*. *umbratica*, with extended flower longevity allowing DAS to occur being advantageous when pollinators are absent, while reduced floral longevity and prevention of DAS being favored when flowers are pollinated by pollinators. Thus, flower longevity in *S. umbratica* varies so as to optimize reproductive output and resource efforts, and is dependent on the availability and effectiveness of pollinators to pollinate flowers.

## Introduction

Delayed autonomous selfing (DAS), which occurs after opportunities for pollination by a pollen-vector have passed, can provide reproductive assurance and for this reason has been termed a “best-of-both-worlds” mating strategy (Goodwillie and Weber, 2018). DAS is expected to be favored by selection when inbreeding depression caused by selfing is >0 and <1 (Lloyd, 1992; Goodwillie and Weber, 2018; Hildesheim et al., 2019). DAS has been reported in 23 orders, 40 families, 56 genera, and 68 species of plants (Chaudhary et al., 2018; Goodwillie and Weber, 2018; Lemos et al., 2020), but for ca. 94% of these species, analyses have not described how floral changes involved in delayed selfing might be terminated if prior, successful and sufficient pollination by a pollen-vector occurs. In fact, DAS has generally been regarded as a process that continues, irrespective of prior pollination by a pollen-vector (Sun et al., 2005; Fan and Li, 2012; Brys et al., 2013; Chaudhary et al., 2018). Furthermore, most previous studies have examined species possessing a large number of ovules per flower and have not assessed the extent to which ovules are fertilized through pollination by insects or as a result of DAS.

Flowering plants exhibit high diversity in floral longevity, often reflecting adaptive responses to prevailing ecological conditions, efficient pollinators, and pollen dissemination/receipt (Ashman and Schoen, 1994; Castro et al., 2008; Roddy et al., 2021). The effects of pollination on floral longevity variation have been evaluated in several plant species, showing, for example, that it may be curtailed after pollination to prevent further expenditure on floral resources (Roddy et al., 2021) and/or to direct pollinators to non-pollinated flowers (Milet-Pinheiro et al., 2015). In contrast, floral longevity may be extended when rain reduces pollen viability, thus increasing the probability of pollinators depositing viable pollen on stigmas and securing high seed set (Domingos-Melo et al., 2020). In some species, flower wilting or abscission have been shown to trigger DAS to increase seed set in the absence of pollinators or when pollen is limited (Costa and Machado, 2017; Domingos-Melo et al., 2018). However, if pollinators are present and cross-pollination is completely successful, flower abscission causing termination of DAS might be expected, so as to reduce floral maintenance costs (Ashman and Schoen, 1994; Hildesheim et al., 2019). This may be especially true in annual herbs where resources allocated to flowers are generally limited, and also in species producing few ovules per flower.

To our knowledge, few studies have evaluated experimentally the possibility of termination of processes leading to DAS following prior successful pollination of flowers by insects. We tested this hypothesis in *Salvia umbratica* Hance (Lamiaceae), an annual herb native to northern China, which produces four ovules per flower. Previous reports suggest that *Salvia* species are self-compatible (Jorge et al., 2014; Barrionuevo et al., 2021), and personal observations show that insect pollinators (bumblebees) frequently visit several flowers consecutively on a plant, and therefore often carry on their bodies a mixture of pollen grains from the same and other plants. A preliminary investigation of flower longevity in a natural population of *S. umbratica* showed that single flowers can remain opening for about 85 h in the absence of pollinators with high levels of fruit and seed set, indicating that delayed selfing occurs in the species. DAS has not been previously reported in Lamiaceae (Goodwillie and Weber, 2018), although there have been suggestions of different levels of autonomous selfing occurring in *Salvia* (Haque and Ghoshal, 1981; Navarro, 1997; Jorge et al., 2014; Rosas-Guerrero et al., 2017; Cuevas et al., 2018; Barrionuevo et al., 2021). Here we report an examination of the flowering process of *S. umbratica* in the presence and absence of pollinators, as well as investigations of fruit and seed set following different pollination treatments. We show that DAS occurs in this species and results in high fruit and seed set, but that floral changes leading to it are terminated by prior successful and sufficient pollination of flowers by a pollinator.

## Materials and Methods

### Species Distribution and Floral Structure

**Figure 1:**
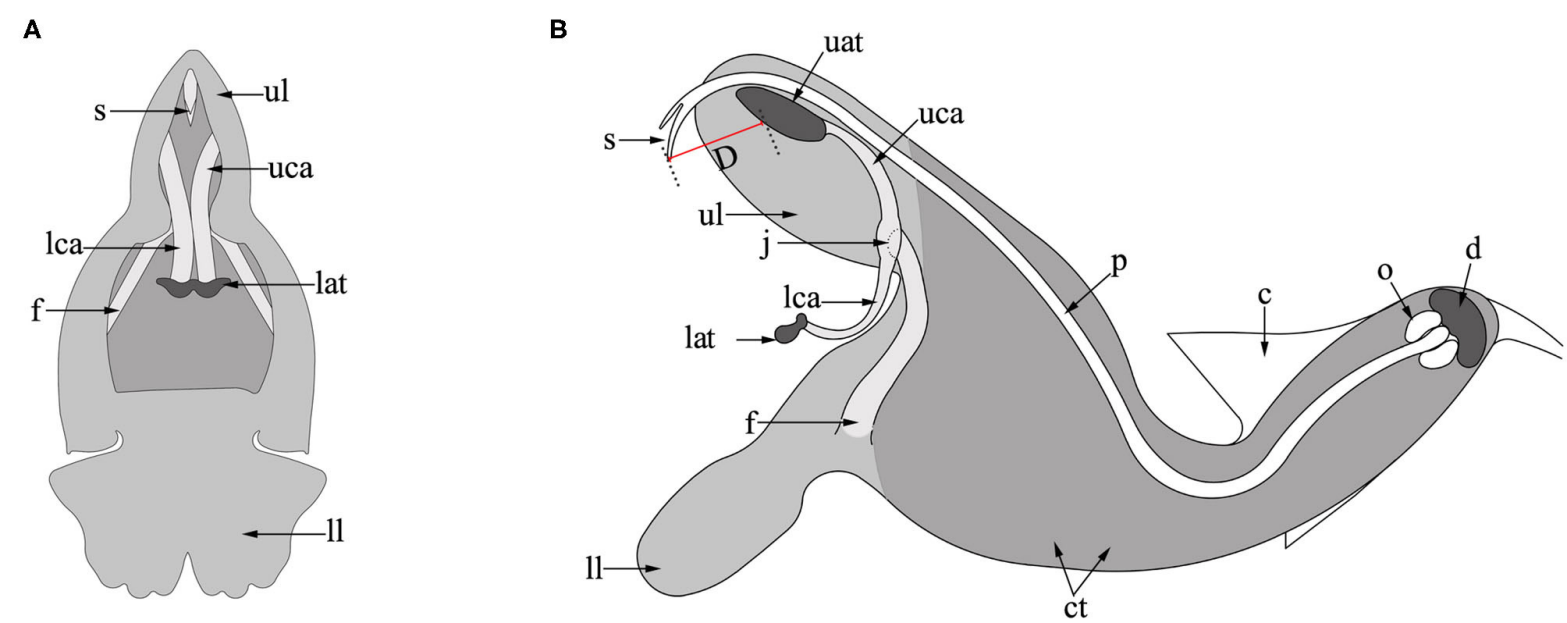
Flower structure of *S. umbratica*. **(A)** Front view; **(B)** lateral view. ct, corolla tube; ul, upper lip; ll, lower lip; uat, upper anther theca; lat, lower anther theca; uca, upper connective arm; lca, lower connective arm; j, joint; f, filament; p, pistil; s, stigma; c, calyx; o, ovary; d, disc; D, distance (between the stigma and upper anther thecae).

*Salvia umbratica* Hance. (Lamiaceae) is an annual herb with erect stems of 1–1.2 m in height. It grows between 600 and 2,000 m a.s.l. on hillsides, and in valleys or roadsides in northern China where it is native to Anhui, Gansu, Hebei, Hubei, Shaanxi, and Shanxi provinces. The type specimen of the species was collected from Beijing (Wu and Li, 1977; Li and Hedge, 1994). The flower of *S*. *umbratica* is typical of *Salvia* being composed of a bilateral symmetrical corolla (Figure 1), the upper lip (hood) of which is formed from two petals, while the lower lip (palate) develops from the remaining three petals. The two stamens are each composed of two anther thecae and two connective arms attached to a filament by a joint (Huang et al., 2014). The upper anther theca (upper theca) of each stamen is positioned in the hood of the corolla just below the style of the pistil and is connected to the lower anther theca (lower theca) via the connective arm. A much larger quantity of pollen is produced in the upper than the lower thecae. In fully developed flowers, the stigma is bifid and protrudes slightly from the upper lip with its receptive surface facing outwards. At the base of the corolla tube is a disc containing a nectary, above this is an ovary containing four ovules (Strelin et al., 2017).

### Study Sites

Field studies were conducted in 2018 and 2020 at the Beijing Forest Ecosystem Research Station (BFERS) located on Dongling Mountain (115°25′33^′′^ E, 39°57′29^′′^ N, elevation 1,200 m) where a semi-humid and monsoon climate prevails, with an average annual temperature of 5–10°C and annual precipitation of 500–650 mm (Xu et al., 2018). The studies included observations on insect pollinators and amount of pollen deposited during a single pollinator visit, the process of DAS in the absence and presence of pollinators, and seed set following various pollination treatments.

To confirm further the occurrence and prevention of DAS and the reproductive fitness of *S. umbratica*, greenhouse studies were conducted at the Shanghai Chenshan Botanical Garden (SCBG) on plants transplanted in May 2020 from the wild at the BFERS. These studies investigated the amount of pollen required for sufficient pollination and high seed set, the prevention of DAS by prior sufficient pollination, and levels of seed set resulting from DAS in the absence of pollinators. Both field and greenhouse studies were conducted in August and September during the natural blooming period of *S. umbratica*.

### Observations on Insect Pollinators and Amount of Pollen Grains Deposited on Stigmas During a Single Pollinator Visit

Pollinator visits to *S. umbratica* flowers were recorded in the field during the natural flowering period on sunny days on the 27, 28, and 31 of August, 2018. For this purpose, we established two 1 ×1 m quadrats at the field site, each of which contained four or five individual plants of *S. umbratica* and an average of 26 and 28 blooming flowers. Two SONY HDR-CX510E cameras were used to record the types, numbers, number of visits, and visit duration of flower-visiting insects in each quadrat between 08:00 and 17:00 each day. We noted the behavior of insect visitors within the quadrats, recording legitimate visits to flowers, i.e., where a pollinator contacted the stigma and stamens while searching for nectar (see Li and Huang, 2009; Ma et al., 2014), and illegitimate visits, i.e., where a pollinator robs nectar and pollen without pollinating the flower. Specimens of pollinators were captured with an insect-net and stored in ethanol (70%) for accurate identification. Vouchers were deposited at the entomological collection at the Horticulture Department of SCBG.

To detect the amount of pollen deposited on a stigma during a single pollinator visit, 45 flower buds were randomly selected from 15 *S. umbratica* plants (3 flowers per plant) that grew naturally at the field site outside the quadrats. Each bud was enclosed in a nylon mesh bag which was removed immediately after the bud opened allowing pollinators to visit the flower produced. After a single visit by a pollinator, the pistil was immediately removed from the flower and pollen deposited on the stigma was counted using a STV-120m portable microscope (Japan, Kenko).

### Process of DAS in Absence and Presence of Pollinators

The process and timing of DAS in flowers of *S. umbratica* were investigated first in the absence of pollinators. Two flower buds from each of 10 plants of *S. umbratica* growing naturally at the field site were placed in nylon mesh bags. Photographs of the development of the stigma and upper thecae of each flower were taken using a Nikon D300s digital camera, and records of the distance, D (see Figure 1B), between the stigma and upper thecae (measured using Photoshop CS3, Adobe, USA), were made when each flower first opened (0 h) and thereafter at 24 h intervals until DAS was completed, i.e., when *D* = 0. Flowers were deemed to have first opened when the upper and lower lips of the corolla mouth had just separated. This experiment was then repeated with minor modifications in the presence of pollinators. Thus, another two flower buds from each of 10 different plants of *S. umbratica* were placed in nylon mesh bags with a record taken of distance between the upper thecae and the stigma when each flower opened (0 h). Bags were then removed from flowers to allow pollinators to visit them. After a pollinator had entered the corolla tube and successfully pollinated a selected flower, stigma-upper thecae distance was measured again and at intervals of 24 h thereafter.

### Fruit and Seed Set Following Different Pollination Treatments at Field Site

To confirm the breeding and mating system characteristics of *S. umbratica*, flowers on a number of different plants in the field were subjected to five different pollination treatments after which fruit and seed set was recorded per flower. These treatments were: (1) autogamy, involving bagging flowers to allow autonomous selfing to occur; (2) simulated geitonogamy, involving hand-pollination of emasculated and bagged flowers using pollen from another flower of the same plant; (3) simulated xenogamy, as in (2) above but using pollen from a different plant; (4) detection of apomixis, involving emasculation and bagging of flowers to determine if seed were produced; and (5) open-pollination, to determine seed set in flowers left to be naturally pollinated. Each of the five treatments was repeated on three flowers on each of 30 plants (90 flowers per treatment), with fruit production and seed set (number of seeds/4 ovules) per flower recorded 3–4 weeks after pollination had occurred.

### Amount of Pollen Required for Sufficient Pollination and High Seed Set

To determine the amount of pollen required for sufficient pollination and fertilization of ovules in a flower, flowers on a number of greenhouse cultivated plants were subjected to five different pollination treatments using a mixture of pollen grains. Pollen was obtained from an equal number of newly dehiscent anthers from a flower on the same plant and a different plant, mixed on parchment and deposited on stigmas using a dissecting needle. The number of pollen grains deposited was determined using a STV-120m portable microscope (Japan, Kenko). The five treatments involved depositing on the stigma of a flower 1–3, 4, 5–10, 11–20, or >20 pollen grains from the mixture. Each treatment was applied to two flowers on each of 10 plants, i.e., 20 flowers per treatment, with fruit production and seed set per flower recorded 3–4 weeks after pollination had occurred.

### Prevention of DAS by Sufficient Prior Pollination

To test the hypothesis that sufficient prior pollination by a pollinator likely prevents DAS from occurring, 40 flowers across 10 greenhouse cultivated plants were hand-pollinated immediately after they opened in either of two ways using a mixture of pollen grains. In 20 flowers, 1–3 pollen grains were deposited on their stigmas, whereas in the other 20 flowers >20 grains were deposited. Stigma-upper thecae distance in flowers (D) was recorded at the stage they were pollinated and at 24 h intervals thereafter. Because *Salvia* produces four ovules per flower, for 20 flowers the number of pollen grains deposited on a stigma was less than the number required to fertilize all ovules (i.e., there was pollen-limitation), whereas for the other 20 flowers the number of pollen grains deposited on the stigma was more than required to fertilize all ovules (i.e., no pollen-limitation; Knight et al., 2005; Jorge et al., 2014).

### Fruit and Seed Set Resulting From DAS in Absence of Pollinators

In a follow-up study, the effect of DAS on seed set was determined on five plants cultivated in a greenhouse in the absence of pollinators. Flower number per plant averaged 174 and ranged between 48 and 244 across the five plants. During the period from the first flower opening to the last flower dropping, we recorded at 24 h intervals the proportion of flowers on a plant that had completed the process of DAS (when stigma-upper thecae distance was zero), and subsequently the fruit and seed set of these flowers.

### Statistical Analyses

All statistical analyses were performed using SPSS 22.0 (Chicago, IL, USA). The analysis of differences in mean stigma-upper thecae distances at different times in flowers pollinated with 1–3 pollen grains was conducted using a generalized linear mixed model (GLMM), with distances as dependent variable, time as fixed effect, and plant as a random factor. A *t*-test was performed to analyze differences of stigma-upper thecae distances at the time of pollination and 24 h later in pollination treatment with >20 pollen grains. Differences in mean fruit set and seed set among different pollination treatments were analyzed by one-way ANOVA followed by a *post-hoc* Tukey's test after excluding treatment 4 (emasculation and bagging of flowers produced no seed indicating an absence of apomixis).

## Results

### Observations on Insect Pollinators and Amount of Pollen Deposited on a Stigma During a Single Pollinator Visit

**Figure 2:**
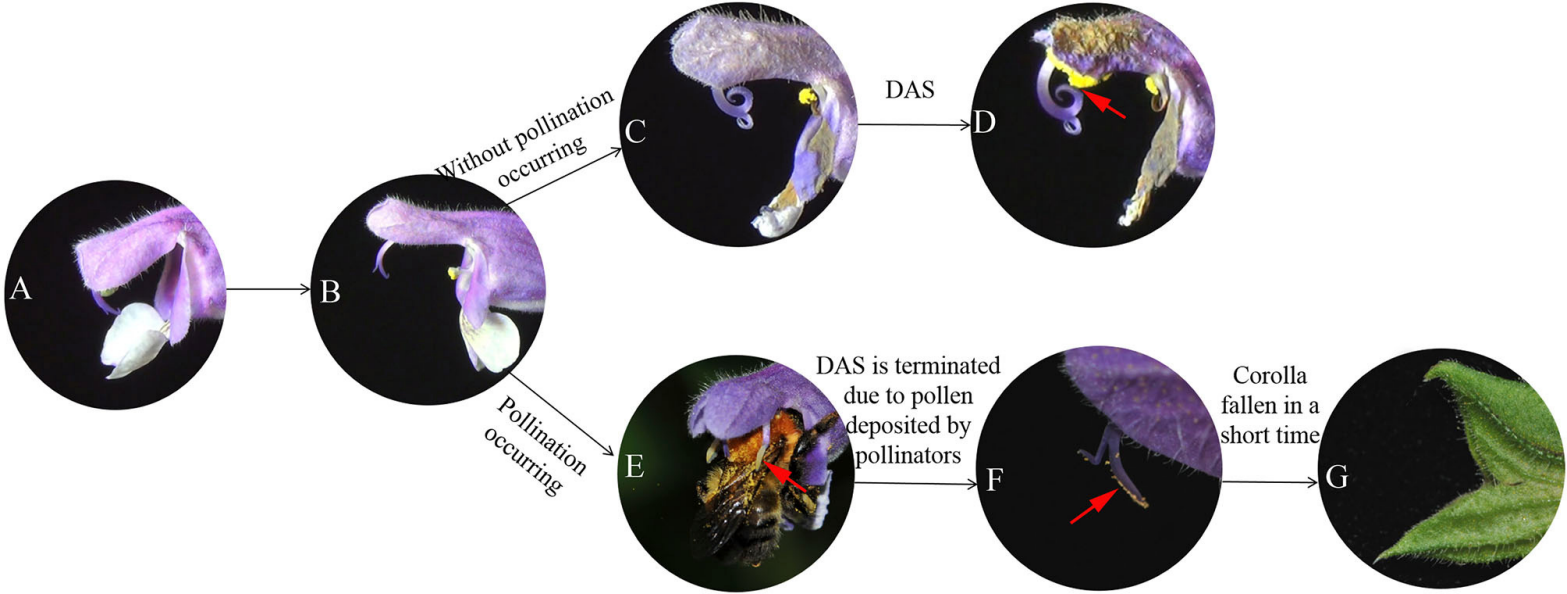
**(A–D)** Different stages of flower development during the process of DAS, and **(E,F)** when DAS is terminated after bumblebee pollination. **(A)** Flower opens when upper and lower lips of corolla separate (0 h); **(B)** fully opened flower shows stigma with receptive surface oriented outward and unfurled; **(C)** style begins to recurve, orienting receptive surface of stigma toward upper thecae; **(D)** receptive surface of stigma makes contact with upper thecae resulting in selfing (arrow shows stigma receptive surface touching upper thecae); **(E)** flower pollinated by a bumblebee; **(F)** style does not recurve toward upper thecae, arrow indicates stigmatic receptive surface has pollen deposited on it by pollinator; **(G)** corolla has fallen away (dropped) from flower a short time after pollination.

Over the 3 days that observations were made, a total of 174 legitimate pollinator visits to flowers in quadrats were recorded. These were by the bumblebees *Bombus opulentus, B*. *longipes, B*. *consobrinus*, and *B*. *hedini*. On entering the corolla tube, bumblebees searched for and fed on nectar. In doing so, pollen on their backs was deposited on the stigma. Furthermore, they pushed against the lower connective arms of the thecae (Figure 1), causing the upper connective arms to bend downwards, resulting in pollen from the upper thecae being deposited on their backs (Figure 2E). On average, the number of visits per flower was 1.23 ± 0.31 per day with visits per flower lasting 2.19 ± 0.09 s.

Counts of pollen grains deposited on the stigma of a flower during a single pollinator visit after the flower opened showed that, on average, 26.31 ± 2.07 (11–65 pollen grains per stigma, *n* = 45) pollen grains were deposited, which is more than enough to result in a high level of fruit and seed set (see below).

### Process of DAS in Absence and Presence of Pollinators

**Figure 3:**
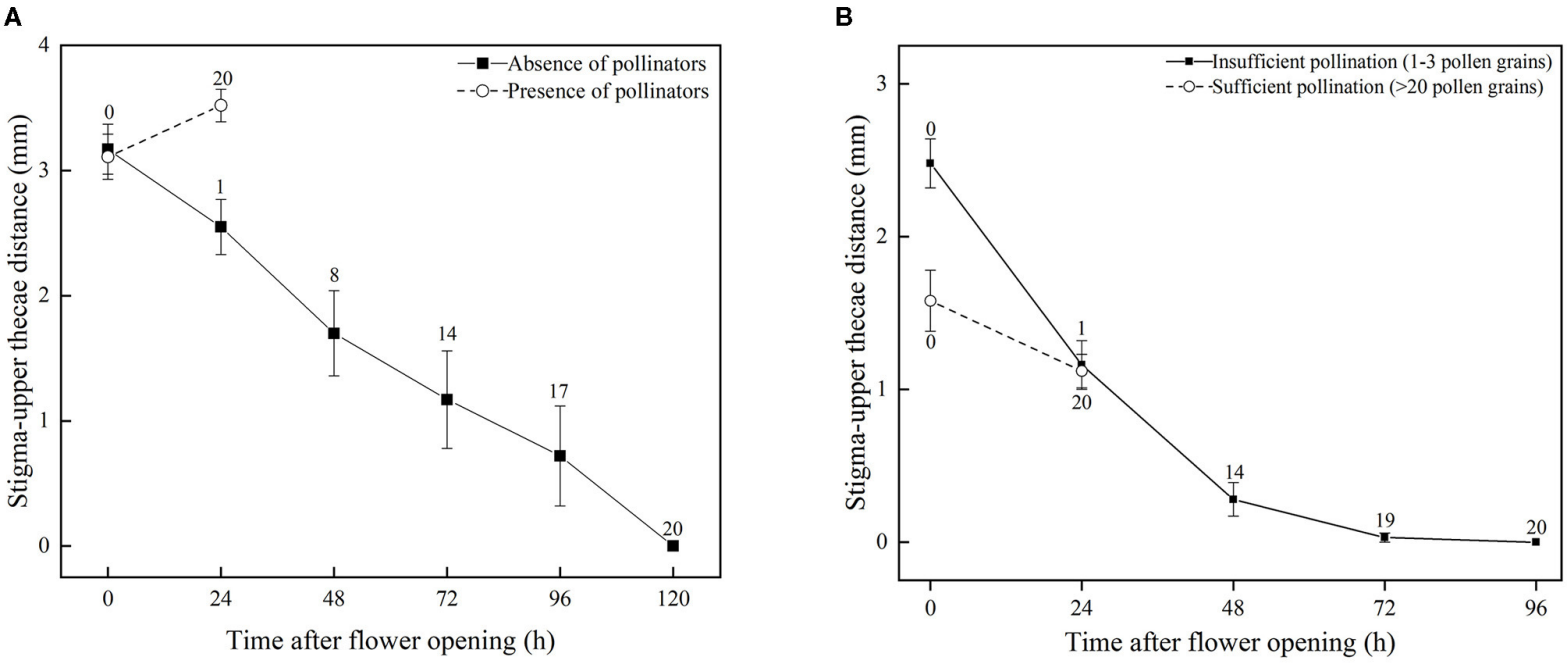
Occurrence and prevention of DAS in *S. umbratica* (bars indicate standard errors). **(A)** Mean stigma-upper thecae distance of flowers in absence of pollinators and also after pollination by a pollinator (presence of pollinators). Records were taken when a flower first opened (0 h) and thereafter at 24 h intervals until pollination was completed in all flowers. Numbers above standard error bars indicate total number of flowers pollinated at given time. **(B)** Mean stigma-upper thecae distance of flowers subjected to insufficient pollination (stigmas hand-pollinated with 1–3 pollen grains) and sufficient pollination (hand-pollinated with >20 pollen grains) treatments. Records were taken of stigma-upper thecae distance when a flower was pollinated with a mix of pollen grains causing either insufficient fertilization or sufficient fertilization (0 h) and thereafter at 24 h intervals. Numbers above standard error bars indicate total number of flowers self-pollinated (stigma-thecae distance, *D* = 0) at given time.

Based on observations made on bagged flowers (Figures 2A–D) and measurements of distance between the stigma and upper anther thecae from the time a flower first opens until DAS is completed (*D* = 0) (Figure 3A), it was evident that DAS occurs in *S. umbratica* when flowers are not pollinated by insects. ANOVA showed that stigma-upper thecae distance decreased significantly with time [*F*_(5,74)_ = 8.808, *p* < 0.001]. At 24 h after a flower first opened the style had begun to recurve causing the receptive stigma surface to become angled toward the upper thecae (Figure 2C). At this stage, mean stigma-anther thecae distance was reduced from the initial mean of 3.17 mm at the time of flower opening (Figure 3A). Recurvature of the stigma toward the thecae continued at different rates in different flowers. In 70% of flowers (14 out of 20), DAS (*D* = 0) was completed by 72 h after the flower first opened. However, in three flowers it took 120 h to complete. On average, DAS took 66.7 h to complete across the 20 flowers examined. Corollas began to fade in color and dry out after styles started to recurve (Figures 2C,D). The entire sequence of events leading to DAS are shown in Supplementary Video 1.

For flowers that were accessible to pollinators, mean stigma-upper thecae distance was 3.11 ± 0.80 mm at time of flower opening (0 h) (Figure 3A). All marked flowers were visited and pollinated by insects (Figure 2E) during the following 24 h (mean time to successful pollination equaled 19.7 h) and by 24 h mean stigma-upper thecae distance was 3.52 ± 0.59 mm (Figure 3A). The two means were not significantly different from each other (*t* = −1.796; *p* = 0.081). The receptive surface of the stigma remained facing outwards from the corolla with large amounts of pollen deposited on it (Figure 2F). The corolla quickly dropped from a flower after a pollinator visited it (Figure 2G) and the floral processes leading to DAS were terminated.

### Effect of Pollination Treatment on Fruit and Seed Set

**Figure 4:**
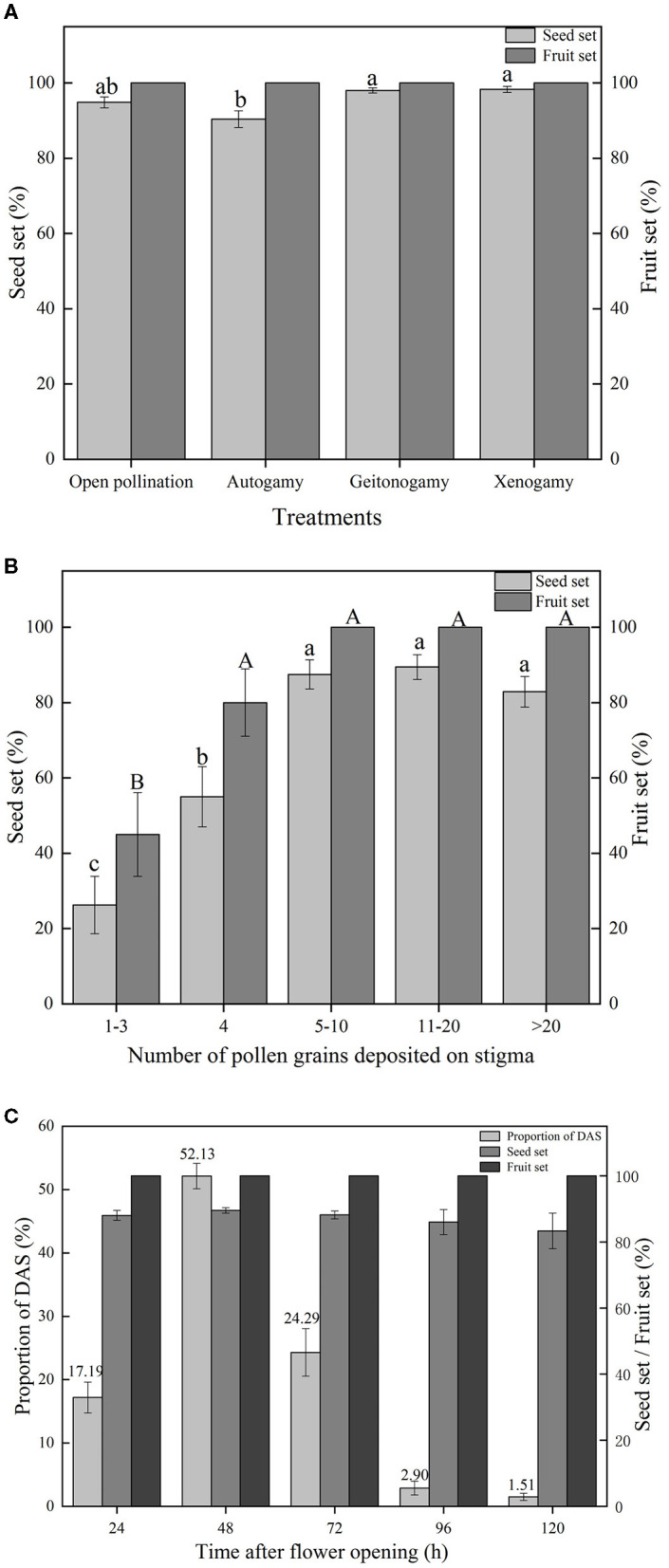
**(A)** Mean fruit set and seed set of flowers subjected to different pollination treatments (bars indicate standard errors). Treatments sharing the same letters placed above bars are not significantly different according to Tukey's test. N.B. Autogamy, Geitonogamy, and Xenogamy refer to treatments where seed was produced after autonomous selfing, hand-pollination with pollen from same plant, and hand-pollination with pollen from a different plant, respectively. **(B)** Mean fruit set and seed set of flowers hand-pollinated with different amounts of pollen. Bars indicate standard errors, with treatments sharing the same letters above bars not significantly different according to Tukey's test. **(C)** Proportion of flowers in which DAS was completed at different times after flower opening. Numbers above standard error bars indicate total proportion of DAS flowers (stigma-upper thecae distance, *D* = 0) at given time. Fruit set and seed set in these flowers is also shown.

No fruits and seeds were produced by emasculated and bagged flowers, and it was concluded, therefore, that apomixis did not occur in the species. Fruit set for all other treatments was 100% (Figure 4A). High levels of seed set were recorded across these other treatments (Figure 4A); however, a one-way ANOVA showed that differences were significant [*F*_(3,324)_ = 7.163, *p* < 0.001]. *Post-hoc* pairwise comparisons of means using Tukey's test indicated that mean seed set of bagged flowers (90.38%, resulting from DAS) was significantly lower than mean seed sets of flowers subjected to either cross-pollination by flowers of the same plant (98.03%, geitonogamy, *p* = 0.002) or different plants (98.29%, xenogamy, *p* = 0.001). However, mean seed set of bagged flowers was not significantly different from that of open-pollinated flowers (94.88%, *p* = 0.098), which, in turn, was not significantly different from the means of the two other treatments. Flowers subjected to the geitonogamy and xenogamy pollination treatments had equivalent high seed set (*p* = 0.999).

### Amount of Pollen Required for Sufficient Pollination and High Seed Set

High levels of fruit set (100%) and seed set (>80%) resulted when the number of pollen grains deposited by hand on a stigma of a bagged flower was 5–10 or greater, but were reduced to 45.00 ± 11.12% and 22.50 ± 6.59%, respectively, when 1–3 grains were deposited [Figure 4B; One-way ANOVA, Fruit set, *F*_(4,95)_= 12.579, *p* < 0.001; Seed set, *F*_(4,95)_ = 20.432, *p* < 0.001]. There was no significant difference for fruit set when 4 or more pollen grains were deposited on a stigma (80 vs. 100%, *p* = 0.235), however seed set increased when >4 grains were deposited (Figure 4B).

### Prevention of DAS by Sufficient Prior Pollination

In flowers in which 1–3 pollen grains were deposited on the stigma, mean stigma-upper thecae distance decreased gradually over time (Figure 3B) in a similar, though more rapid, way to when pollinators were excluded from flowers (and presumably zero pollen grains were deposited on stigmas) (Figure 3A). Thus, DAS was completed in 14 of the 20 flowers by 48 h after they opened, and in all flowers by 96 h, and on average took 55.2 h to complete. This average time to completion was not significantly different to that (66.7 h) estimated for bagged flowers where access by pollinators was prevented (*t* = 1.807, *df* = 38, *p* = 0.079). In marked contrast, DAS did not occur in flowers in which >20 pollen grains were deposited on stigmas. Mean stigma-upper thecae distance in these flowers remained >1 mm at 24 h after they were pollinated, and was not significantly different from D at the time stigmas were pollinated (Figure 3B; *t* = 2.00; *p* = 0.053). After 24 h, the corollas of these flowers quickly dropped, indicating that sufficient prior pollination and fertilization had occurred preventing DAS from occurring.

### Fruit and Seed Set Due to DAS in Absence of Pollinators

Records made on bagged flowers of five plants showed that DAS was completed after flower opening in ~17% of flowers by 24 h, a further 52% of flowers by 48 h, and another 24% by 72 h (Figure 4C). Thus, DAS was completed in ~94% of flowers by 72 h after opening, with only a small proportion of flowers taking longer to complete this process. There was no significant difference in fruit set (all 100%) and seed set (always >80%) of flowers in which DAS was completed at different times over the 24–120 h period that records were taken [Figure 4C; One-way ANOVA, seed set, *F*_(4,765)_ = 0.813, *p* = 0.517].

## Discussion

*Salvia* species have an effective floral mechanism for promoting cross-pollination (Claßen-Bockhoff et al., 2003, 2004) and previous studies have shown that bumblebees which pollinate most of these species carry large pollen loads (up to 400 pollen grains) on their body parts (Celep et al., 2014). Our results show that a minimum of 5–10 pollen grains per stigma (i.e., 1–2 pollen grains per ovule) is required to achieve high seed set in *S. umbratica* and that on average, 26 pollen grains per stigma (range 11–65) are deposited by a single pollinator (bumblebee) visit in the field (representing the average of four bumblebee species operating as pollinators). This is more than enough pollen to ensure sufficient pollination and high seed set. However, in the absence of pollinators, we showed that delayed autonomous selfing (DAS) occurs in *S. umbratica* as a result of style recurvature, causing the receptive surface of the bifid stigma to come into contact with the upper anther thecae so that self-pollen is deposited upon it. This is the first demonstration of DAS occurring in the Lamiaceae, despite considerable previous and current interest in the pollination biology of *Salvia* (Celep et al., 2014; Cairampoma et al., 2020; Drew, 2020; Barrionuevo et al., 2021). Our results also showed that a single bumblebee visit to a flower prevents DAS from occurring, because it results in sufficient cross-pollination and fertilization, causing the flower to drop before the style recurves.

For ca. 82% of species (56/68) reported to exhibit DAS, the number of ovules per flower is greater than the four present in *S. umbratica* (Chaudhary et al., 2018; Goodwillie and Weber, 2018; Lemos et al., 2020). Theoretical models predict that stochastic variation in floral mating success creates an advantage to producing many ovules per flower because a plant will often gain greater fitness from occasional abundant seed production in randomly successful flowers than it loses in resource commitment to less successful flowers (Burd et al., 2009). However, species with high numbers of ovules per flower may frequently experience pollen-limitation, i.e., insufficient pollen is deposited on the stigmas of their flowers during one or several visits by a pollinator to fertilize all ovules. In these species, DAS might be advantageous and be expected to continue after flowers have been pollinated by a vector, so as to increase the amount of pollen deposited on stigmas and the probability of all ovules being fertilized. Supporting evidence for this comes from the genus *Centaurium*, in which species commonly produce flowers containing >200 ovules. Brys and Jacquemyn (2011) showed that though stigmas of these flowers are receptive to foreign pollen on the first day a flower opens, during the second and third days after opening, the anthers curl toward the stigma to bring about delayed selfing. In addition, in *Hedychium yunnanense* which produces ~60 ovules per flower, it has been shown that floral processes continue after hand cross-pollination to bring about contact between stigmas and anthers so as to effect selfing (Ma et al., 2012). Of course, if during a single pollinator visit sufficient pollen is deposited to fertilize all ovules of a species that produces large numbers of ovules per flower, there would seem no advantages to having a DAS mechanism except in environments where pollinators were rare or absent. In the Orchidaceae and subfamily Asclepiadaceae, for example, deposition of a single pollinium containing millions of pollen grains is more than sufficient to fertilize all ovules of a flower (Johnson and Edwards, 2000). However, even in these plant groups some species have mechanisms enabling selfing to occur should cross-pollination fail (Yamashiro and Maki, 2005; Tałałaj et al., 2019).

Species such as *S. umbratica*, which produce one or a few ovules per flower, are unlikely to experience pollen-limitation following a single visit by a pollinator. However, the occurrence of DAS in these species should be favored if they occur in environments where pollinators are often rare or absent. This may be the case for *S*. *umbratica*, which has spread to northern and eastern parts of China far away from where most other members of subg. *Sclarea* Benth. occur in the Hengduan Mountains and Himalayas. Our studies show that floral longevity in *S. umbratica* is highly variable and dependent on whether or not flowers are cross-pollinated. If flowers are cross-pollinated soon after opening, floral longevity is cut short, with corollas dropping within 24 h of a single bumblebee visit. In contrast, when flowers remain unpollinated, floral longevity is extended allowing DAS to take place. The ability to reduce quickly the longevity of a flower and terminate DAS following cross-pollination will allow resources used in maintenance of floral structures to be diverted for other purposes and should be favored if this leads to an increase in fitness of an individual. This might be particularly important in an annual herb like *S. umbratica* with limited resources available for reproduction.

Some interesting comparisons can be drawn between our findings for *S. umbratica* and those for *Kosteletzkya virginica* (Malvaceae), a herbaceous species native to North America that was introduced to China <20 years ago. Although we are unaware of any reported data regarding floral characteristics and reproductive biology of *K. virginica* in areas where it is native, the species has been shown to exhibit DAS in its introduced range (Ruan et al., 2008). Like *S. umbratica*, it possesses a low number of ovules (five) per flower but in contrast has a five-lobed stigma. Pollination of these stigma lobes prevents their recurvature and therefore terminates floral processes leading to DAS (Ruan et al., 2008). However, recurvature of each stigma lobe is independent of that of other stigma lobes; thus, if only one lobe is pollinated it remains erect, whereas each of the four adjacent unpollinated lobes recurve. Ruan et al. (2008) showed that pollination of a stigma lobe by only one pollen grain prevents recurvature of the lobe provided that a pollen tube is produced which grows beyond the stigma lobe. In contrast, we found that in *S. umbratica* pollination by >4 pollen grains is required to prevent style recurvature. Ruan et al. (2008) further demonstrated that pollen tube growth rather than pollen quantity is the signal that halts stigma lobe curvature in *K. virginica*. It will be interesting to establish in future work if the same is true for style recurvature in *S. umbratica*.

A further difference between *K. virginica* and *S. umbratica* concerns the speed at which recurvature of stigma lobes or styles occurs. Whereas, unpollinated stigma lobes of *K. virginica* begin recurving within 1 h of a flower opening and contact with anthers is made after a further 8–10 h (Ruan et al., 2008), style recurvature is much slower in *S. umbratica*, such that in flowers not accessed by pollinators or hand-pollinated with only 1–3 pollen grains per stigma, contact between the stigma and upper anther thecae occurred on average ca. 55–67 h after they opened. Why the process of delayed selfing is completed much more quickly in *K. virginica* than in *S. umbratica* is an interesting question. Flowers remain open for only a day in *K. virginica* but for several days in *S. umbratica*. It is possible that pollinators are more plentiful in habitats where *K. virginica* occurs compared with those occupied by *S. umbratica*, and that cross-pollination is normally completed very soon after a flower of *K. virginica* opens. If this is the case, it might be expected that selection has favored reduced flower longevity in *K. virginica*. In support, Castro et al. (2008) showed that in *Polygala vayredae* flower longevity varied in response to the abundance of efficient pollinators indicating that increased longevity might maintain the opportunity for cross-pollination and fertilization in this species when pollinators are scarce. However, there are many factors that can influence flower longevity (Ashman and Schoen, 1994; Costa and Machado, 2017; Domingos-Melo et al., 2018; Roddy et al., 2021) and further research is required to establish why non-cross-pollinated flowers of *S. umbratica* remain open for a much longer time than those of *K. virginica*.

Termination of floral processes resulting in delayed selfing, following prior pollination by a pollen-vector, might not be uncommon, although as far as we are aware it has only been demonstrated previously in Malvaceae (Buttrose et al., 1977; Klips and Snow, 1997; Ruan et al., 2004, 2008; Seed et al., 2006). There are indications, however, that it also occurs in self-compatible Asteraceae. For one such species, *Senecio vulgaris*, it has been shown that florets (each containing a single ovule) often produce elongated styles with no or little self-pollen on their bifid stigmas (Irwin et al., 2016; Love et al., 2016). If stigmatic lobes of these styles are not pollinated by a pollen-vector, they are thought to recurve and pick up pollen from within the floret below. This process is terminated by prior pollination with either self- or cross-pollen, after which styles quickly shrivel before stigmatic lobes recurve.

In view of the findings of the present study, those in Malvaceae, and the possibility that DAS is terminated in self-compatible Asteraceae when prior, vector-mediated pollination occurs, future studies should determine how widespread termination of DAS is in plants, particularly in species that produce few ovules per flower.

## Data Availability Statement

The raw data supporting the conclusions of this article will be made available by the authors, without undue reservation.

## Author Contributions

Y-KW and Y-PM designed the research. H-WX, Y-BH, Y-HC, and YC performed the experiments. H-WX analyzed the date and wrote a preliminary version of the manuscript. RA, Y-KW, and Y-PM revised the manuscript. All authors read and approved the manuscript.

## Conflict of Interest

The authors declare that the research was conducted in the absence of any commercial or financial relationships that could be construed as a potential conflict of interest.

## Publisher's Note

All claims expressed in this article are solely those of the authors and do not necessarily represent those of their affiliated organizations, or those of the publisher, the editors and the reviewers. Any product that may be evaluated in this article, or claim that may be made by its manufacturer, is not guaranteed or endorsed by the publisher.
